# Neurofibromatosis type 2 initially presenting as a preauricular mass: a case report

**DOI:** 10.1186/s40463-020-00438-1

**Published:** 2020-06-26

**Authors:** Wei-Che Lan, Yu Aoh, Rui-Yun Chen, Hui-Chi Tien, Chia-Der Lin

**Affiliations:** 1 Department of Otolaryngology Head and Neck Surgery, China Medical University Hospital, No.2, Yude Rd., North Dist., Taichung City, 404 Taiwan, Republic of China; 2 Department of Neurology, China Medical University Hospital, No.2, Yude Rd., North Dist., Taichung City, 404 Taiwan, Republic of China; 3 Department of Pathology, China Medical University Hospital, No.2, Yude Rd., North Dist., Taichung City, 404 Taiwan, Republic of China; 4 Department of Otolaryngology Head and Neck Surgery, Asia University Hospital, No. 222, Fuxin Rd., Wufeng Dist., Taichung City, 413 Taiwan, Republic of China; 5 School of Medicine, China Medical University, Taichung, Taiwan

**Keywords:** Neurofibromatosis type 2 (NF2), Schwannoma, Atypical presentation, Preauricular mass, Case report

## Abstract

**Abstract:**

Neurofibromatosis type 2 (NF2) is a rare genetic disease involving multiple tumors of the central and peripheral nervous systems. Most patients with NF2 have bilateral vestibular schwannomas; nonvestibular schwannomas may also develop. While the majority of patients may present with hearing impairment, tinnitus, dizziness and balance disorders, some may present with cutaneous manifestations. We describe the case of a 20-year-old man who initially presented with a solitary subcutaneous painless nodule in the left preauricular area without any other symptoms. He received excisional biopsy for the preauricular mass and the pathologic diagnosis was schwannoma. Magnetic resonance imaging of brain and neck revealed multiple mass lesions over the bilateral cerebellopontine angle cisterns, extending to the bilateral internal auditory canals, bilateral cervical neuroforamens, cervical and upper thoracic spinal canals, and left posterior neck. The patient was diagnosed with NF2 according to the clinical criteria. He underwent gamma knife stereotactic radiosurgery for bilateral vestibular schwannomas and is now under regular monitoring.

**Conclusion:**

NF2 patients may present with an isolated solitary cutaneous schwannoma with no other associated clinical findings. Further assessment is thus warranted in young patients presenting with a peripheral schwannoma despite absence of other clinical findings.

## Background

Neurofibromatosis type 2 (NF2) is a rare autosomal dominant genetic disease involving multiple tumors of the central and peripheral nervous systems, as well as ocular and dermatologic manifestations. The estimated incidence of NF2 is 1 in 33,000 people globally. Mutations in the tumor suppressor gene *NF2* on chromosome 22 that encodes the protein product merlin (or schwannomin) causes NF2 [1]. More than half of all NF2 cases are due to de novo gene mutations that are not inherited from family members [2]. Although bilateral vestibular schwannomas are the most characteristic features of NF2, schwannomas also appear in other peripheral, cranial and spinal nerves. Tumors including meningiomas, gliomas, ependymomas and neurofibromas are also related to NF2 [3]. The diagnosis of NF2 is mostly based on Manchester criteria [4]. However, the presence of a *NF2* gene mutation is not mandatory for the diagnosis.

Cutaneous tumors may prompt further investigations that lead to the diagnosis of NF2, but skin manifestations in NF2 are much less obvious than those associated with neurofibromatosis type 1 (NF1). Of around 70% of patients with NF2 who develop skin tumors, only 10% have more than 10 skin tumors [2]. Schwannomas comprise the majority of cutaneous tumors in NF2 [5]. Multiple schwannomas are more commonly connected with neurocutaneous genetic syndrome and may trigger further evaluation and genetic testing. However, it is not standard practice to perform in-depth investigations for patients presenting with a solitary schwannoma. It has been pointed out that failing to undertake further evaluations may delay the diagnosis and management of diseases such as NF2 [6]. To the best of our knowledge, this is the first report of a patient presenting with an isolated cutaneous schwannoma as the only manifestation of NF2.

## Case presentation

**Fig. 1:**
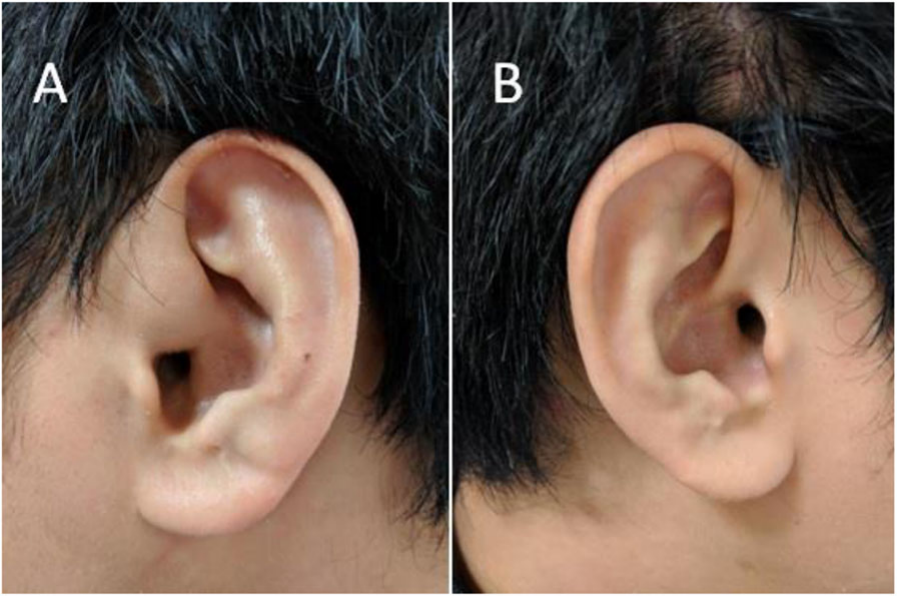
**a** A subcutaneous oval-shaped mass in the left preauricular area. **b** The normal right ear

A 20-year-old male visited our clinic with a chief complaint of a painless nodule in the left preauricular area. He stated that the nodule had been apparent for about 1 month without progressing in size. He had a medical history of sudden onset of right hearing impairment at 15 years of age, but he did not seek medical attention, because the hearing impairment resolved spontaneously 3 days after symptom onset. His family history was unremarkable. Physical examination revealed a painless, subcutaneous, oval-shaped mass (2 × 1.5 cm) in the left preauricular area, in close proximity to the ascending helix of the ear (Fig. 1). There was no preauricular pit on examination. When palpated, the mass showed firm-elastic consistence and limited mobility. No skin lesions or other palpable masses were noted. He had normal external auditory canals and tympanic membranes. Pure tone and speech audiometry tests confirmed that the patient had hearing levels within the normal range. The initial clinical diagnosis was a benign soft tissue tumor.

**Fig. 2:**
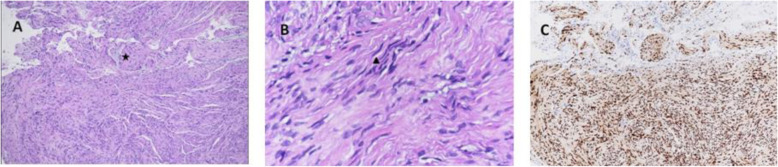
Photomicrographs with hematoxylin and eosin staining (**a, b**) showing a specimen mainly composed of proliferative bundles of nerve-like spindle cells with a focal myxoid substance (★) and wavy nuclei (▲). Immunohistochemical staining was positive for SOX-10 (**c**)

He underwent excisional biopsy for the preauricular mass under local anesthesia. Gross examination revealed that the tumor was encapsulated, firm and gray in color. Microscopically, the specimens showed proliferative bundles of nerve-like spindle cells with a focal myxoid substance (Fig. 2a) and wavy nuclei (Fig. 2b). Nuclear pleomorphism was mild and there was no evidence of necrosis or mitotic figures. Immunohistochemical staining was positive for SOX-10 (Fig. 2c). The pathologic diagnosis was schwannoma.

**Fig. 3:**
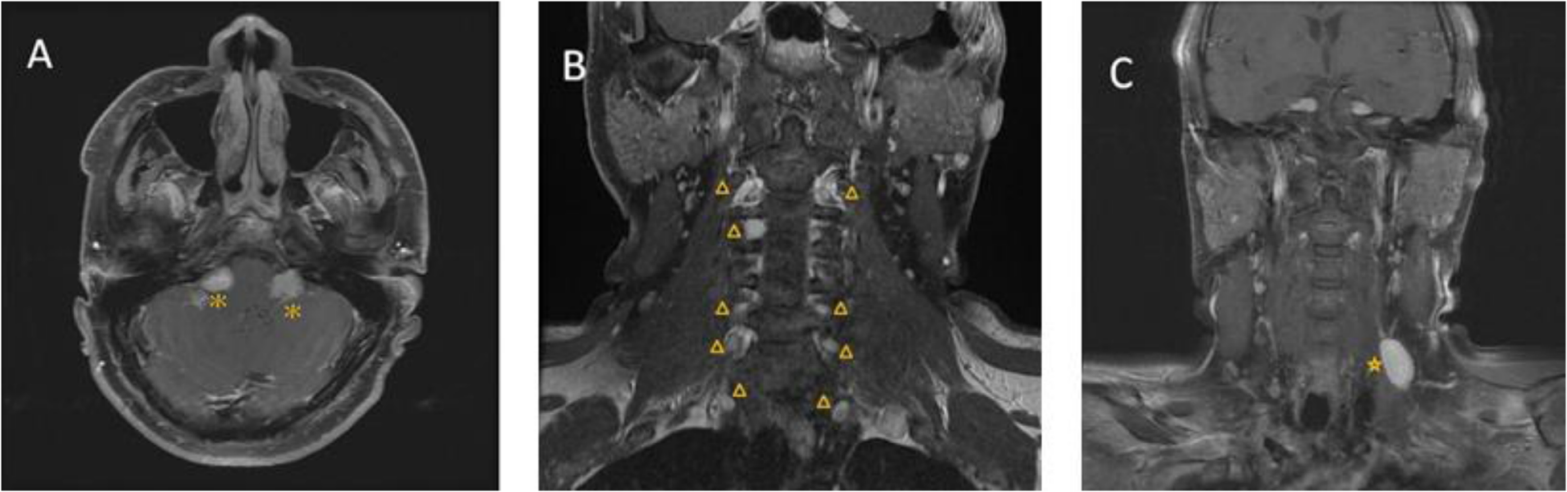
T1-weighted MRI with gadolinium enhancement (**a**: axial view, **b, c**: coronal views) showed bilateral vestibular schwannomas (*), multiple tumors over the bilateral cervical neuroforamens (△) and left posterior cervical neck (★)

Considering the medical history of right sudden hearing impairment and solitary schwannoma in this young adult, magnetic resonance imaging (MRI) was performed. A brain and neck MRI revealed multiple and heterogeneous enhancing lesions including bilateral involvement of the cerebellopontine angle cisterns with extension into the internal auditory canals (Fig. 3a), bilateral trigeminal nerves, the left perimedullary region with extension to the left jugular foramen, bilateral cervical neuroforamens (Fig. 3b), cervical and upper thoracic spinal canals, left posterior cervical neck (Fig. 3c), and the left first intercostal space. The patient was diagnosed with NF2 according to the clinical criteria. He was referred to the neurology clinic, where clinical neurologic examinations revealed no focal neurologic deficits. Subsequent screening for mutations of the *NF2* gene by polymerase chain reaction and direct sequencing identified a heterozygous mutation in the *NF2* gene. The patient underwent gamma knife stereotactic radiosurgery with a prescription dose of 12 Gy for bilateral vestibular schwannomas and is now under regular monitoring by the neurosurgeon.

## Discussion

Most adult patients with NF2 present with unilateral hearing loss as the initial clinical manifestation [3]. Accompanying tinnitus or tinnitus preceding hearing loss may also be reported. Less common symptoms at initial presentation include seizures, headache, muscle weakness, paresthesia, and cutaneous tumors [7]. Cutaneous lesions are not included in the diagnostic criteria for NF2 and, unlike skin features in NF1, are not often addressed [8]. However, cutaneous lesions are fairly common in NF2 patients. In their 2009 review of the clinical manifestations of NF2, Asthagiri and colleagues reported that skin tumors occur in 59–68% of patients with NF2 and include skin plaques, subcutaneous and intradermal tumors [5]. They also noted that subcutaneous tumors and skin plaques are present in up to 48% of patients; intradermal tumors are less frequent [5].

Patients presenting with multiple cutaneous schwannomas and other characteristic symptoms may encourage the physician to conduct further workup. However, our patient presented with a solitary cutaneous schwannoma without other typical features, so was at potential risk of a delayed diagnosis. It is not standard practice to undertake further assessments such as an MRI for patients presenting with a solitary schwannoma, which results in a challenging diagnosis of NF2.

A previously published report describes a case of solitary cutaneous plexiform schwannoma in the left preauricular region associated with NF2 [9]. However, that patient exhibited other symptoms and signs including muscle weakness, paresthesias, progressive hearing loss, and multiple café-au-lait spots, which alert physicians to a genetic syndrome [9]. Schwannomas are mostly sporadic and solitary and are more typically seen in the elderly; such tumors are rare in children and young adults, although one study has reported that almost one-third (29%, 44/153) of patients in a cohort who presented with a solitary schwannoma before the age of 25 years had a causative predisposing gene mutation [6]. This suggests that young people presenting with solitary schwannomas need further workup.

In this case, a brain MRI was performed in order to rule out central tumors, given the rarity of an isolated solitary cutaneous schwannoma in young adult, as well as the patient’s medical history of sudden hearing loss in the past. Diagnosis and management of NF2 would potentially have been delayed if no further imaging studies or surveys had been performed after the excision of the isolated preauricular schwannoma. We hope in future to have data from a larger pool of patients in our institution, to enable us to conduct a cost analysis of genetic screening versus MRI and issue a recommendation as to the most appropriate initial investigation for patients under 20 years of age presenting with an isolated cutaneous schwannoma.

## Conclusion

Based on findings in the current case, the authors recommend that younger adults presenting with a solitary cutaneous schwannoma undergo further investigations, including MRI scans of the brain and spine, to exclude the possibility of multiple tumors.

## Data Availability

Not applicable.

## Abbreviations

NF2: Neurofibromatosis type 2
NF1: Neurofibromatosis type 1
